# Hybrid energy-temperature method (HETM): A low-cost apparatus and reliable method for estimating the thermal capacity of solid–liquid phase change material for heat storage system

**DOI:** 10.1016/j.ohx.2023.e00496

**Published:** 2023-12-02

**Authors:** La Ode Mohammad Firman, Dwi Rahmalina, Ismail, Reza Abdu Rahman

**Affiliations:** Department of Mechanical Engineering, Faculty of Engineering, Universitas Pancasila, Srengseng Sawah, Jagakarsa, DKI, Jakarta 12640, Indonesia

**Keywords:** Heat capacity, Latent heat of fusion, PCM, Phase transition, State of charge

## Abstract

Estimating the total thermal capacity for phase change material (PCM) as heat storage material is extremely important to set the proper operational parameter of the latent storage tank (LST) unit. However, estimating the total thermal capacity for solid–liquid PCM is relatively complex due to temperature-dependent properties for each phase. Thus, predicting the state of charge (SoC) indicator for the LST unit is technically complex. The common approach is taken by estimating the heat of fusion during the melting process and monitoring the working fluid temperature, which makes the SoC estimation less accurate. The present project proposes a reliable method with an affordable apparatus to estimate the total thermal capacity simultaneously based on the temperature of low-temperature PCM. Moreover, sensible heating during phase transition is also estimated precisely based on the energy balance at a given temperature. The developed apparatus employs widely available components at an affordable cost which is possible for further customization. Validation and characterization are done comprehensibly by comparing the measurement from differential scanning calorimetry and previous studies. The results indicate a suitable estimation for estimating the solid–liquid heat capacity, including partial heat capacity and latent heat of fusion.

## Graphical abstract

**Figure:**
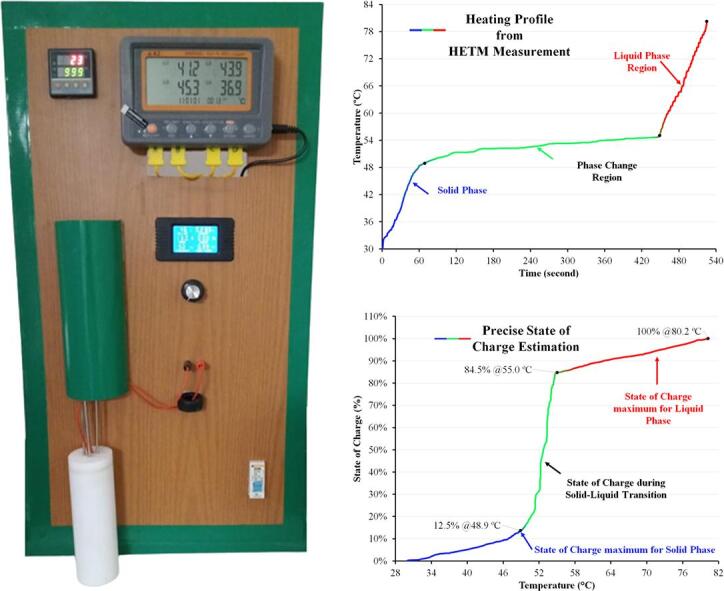

**Specification Table**.

**Table:** 

Hardware name	Hybrid energy-temperature method (HETM): a low-cost apparatus and reliable method for estimating the thermal capacity of solid–liquid phase change material for heat storage system
Subject area	• Mechanical and thermal engineering • Educational tools as a laboratory equipment for heat capacity measurement
Hardware type	• Measuring physical properties and in-lab sensors
Closest commercial analog	Differential scanning calorimetry
Open-source license	Creative Commons Attribution-ShareAlike
Cost of hardware	USD $ 532
Source file repository	Within the article

## Hardware in context

Phase change material (PCM) for latent thermal energy storage (LTES) system offers a higher storage density compared to sensible thermal energy storage (STES). It can be seen in the outstanding improvement of the installed capacity for LTES in heating sectors [1]. The low-temperature application (below 100 °C) utilizes low-cost organic PCM, which is compatible with various storage tank materials [2]. It makes applying organic PCM more attractive to provide a reliable and cost-effective thermal storage system. Material modification for the PCM using high conductivity material [3], modification of the working fluid [4], and container inclination [5] are considered as suitable method to minimize the drawbacks of organic PCM in the LTES system. Thus, current development is focused on improving the operational aspect of the LTES system.

**Fig. 1:**
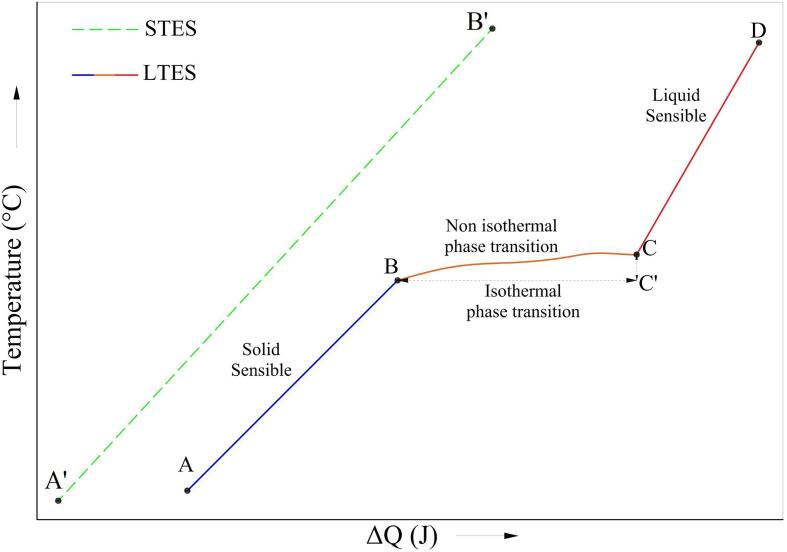
Temperature vs energy curve for typical STES and LTES system.

Continuous effort is addressed to increase the technical parameter and operational protocol for the LTES system, especially for estimating the state of charge (SoC) percentage [6]. The SoC percentage is an essential parameter for the storage system, particularly for the active system which uses working fluid [7], [8], [9], to indicate the total energy stored within the system, similar to the indicator percentage for the electric battery. It makes the operation mode can be adjusted precisely (charging/discharging). For example, Fig. 1 displays the typical temperature/energy curve for the charging operation of the STES and LTES systems. The total energy for STES depends on the heat capacity of the storage material and the working temperature range (A’→ B'). Thus, the SoC can be estimated easily using temperature as the working indicator.

The challenge for estimating the total energy in LTES is the involvement of three consecutive thermal stages from temperature A → D which depends on heat capacity (for sensible heating) and latent heat of fusion (melting process). The specific heat capacity and melting enthalpy can be measured using calorimetry and temperature history methods (THM) [10]. The calorimetry method (differential scanning calorimetry) offers a versatile measurement with a shorter time at a limited sample quantity (less than 1 g). The restricted sample capacity can be solved using THM, which uses a larger sample capacity [11], [12], [13]. However, the measurement uses temperature as the main reference. Thus, it requires complex mathematical analysis to estimate melting enthalpy and specific heat capacity. It makes the actual thermal capacity relatively hard to predict as a dynamic value, leading to complexity in determining the SoC percentage [14].

Estimating the actual thermal capacity using the energy content within the measured PCM can be taken as the ideal approach. It provides a better quantity estimation for the LTES system [15]. It can be done using the working fluid temperature [16], partial charging approach [17], and temperature-enthalpy relation [18]. Also, it can be combined using DSC to estimate the melting enthalpy of the PCM. The approach has a certain advantage but depends on each system configuration, which varies from one type to another. Thus, the estimation of the energy content within the PCM remains unsolved.

The problem related to the actual thermal capacity of the PCM requires a practical approach to be solved for estimating the three-stage heat capacity (solid sensible, phase transition and liquid sensible). Therefore, the present work proposes an affordable apparatus to estimate the actual thermal capacity for low-temperature PCM. The proposed apparatus is a combined calorimetry and THM method. The simplified equation and well-defined instrumentation can be taken as the main advantage of the proposed method. We believe the approach is suitable for practical consideration to estimate the actual thermal capacity of PCM in the LTES system. Also, the components are relatively low cost, which makes the researcher and universities student, especially from low-middle income countries, may develop the apparatus to study and contribute to the development of the LTES system.

## Hardware description

**Fig. 2:**
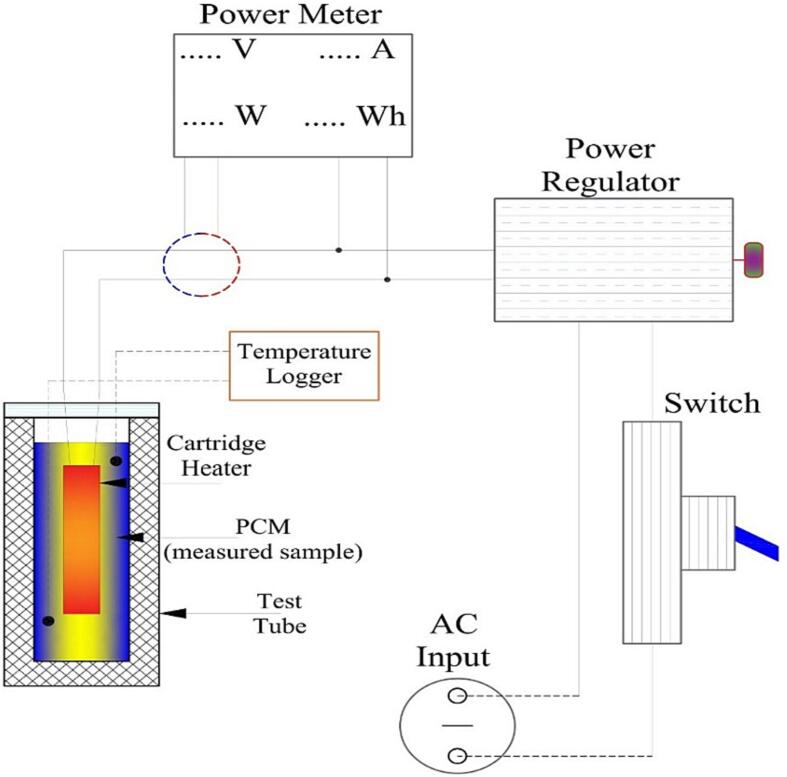
Basic configuration for HETM apparatus.

Fig. 2 presents the apparatus hybrid energy-temperature method (HETM) concept. The thermoelectric conversion occurs within the test tube where the sample is located. The heater is directly in contact with the sample. Thus, the heat generated by the heater can be absorbed by the sample. The power regulator can adjust the heating rate to accommodate flexible measurements with different quantities and types of PCM. The power meter measures the amount of electric energy that flows through the heater. Thus, the relation between temperature increment and energy balance can be set precisely based on the measured energy to the heater and temperature of the sample.

Solid-liquid PCM works between temperature A to Temperature D (Fig. 1). The sensible heating region occurs between A–B (solid sensible) and C–D (liquid sensible). The heat energy for the solid and liquid regions depends on the specific heat capacity, mass and temperature of the PCM. The phase transition region theoretically occurs as an isothermal phase transition (B–C') without a temperature gradient. Thus, the total heat energy from temperatures A–D can be found using the following formula [19]:

(1) $$\Delta Q_{\mathrm{total}}=\left(m∙c_{p,solid}∙\Delta T_{\mathrm{BA}}\right)+\left(m∙\Delta H_{\mathrm{BC}\prime }\right)+\left(m∙c_{p,liquid}∙\Delta T_{\mathrm{DC}}\right)$$

with *m* is the mass of PCM (gram), *c_p_* is heat capacity (J·g^−1^·°C^−1^) at the given state, and Δ*H_BC_*_'_ is the isothermal latent heat of fusion (J·g^−1^). Unfortunately, the actual process indicates the non-isothermal phase transition during solid–liquid transition (B–C) [20], [21], [22]. Thus, two heating stages exist simultaneously: sensible heating and fusion process. The total heat energy during the non-isothermal phase transition can be found using [19]:

(2) $$\Delta Q_{\mathrm{phasetransition}}=\left(m∙c_{p,partial}∙\Delta T_{\mathrm{BC}}\right)+\left(m∙\Delta H_{\mathrm{BC}}\right)$$

Eq. (2) combines the sensible heating and melting process during a non-isothermal phase transition commonly found in the actual LTES system. The term partial specific heat capacity is employed as heat capacity during non-isothermal phase transition. Therefore, the actual thermal capacity for the solid–liquid PCM at specific temperature operation (A–D) can be obtained from [19]:

(3) $$\Delta Q_{\mathrm{total}}=\left(m∙c_{p,solid}∙\Delta T_{\mathrm{BA}}\right)+\left(m∙c_{p,partial}∙\Delta T_{\mathrm{BC}}\right)+\left(m∙\Delta H_{\mathrm{BC}}\right)\;+\left(m∙c_{p,liquid}∙\Delta T_{\mathrm{DC}}\right)$$

The heat capacity (solid, partial and liquid) and latent heat of fusion can be found from the measurement through the HETM apparatus. It allows for estimating the SoC percentage at a specific temperature. Since the energy quantity can be obtained precisely, we can predict the specific SoC in the LTES system by using the proposed equation:

(4) $$\mathrm{SoC}=\frac{\Delta Q_{T_{x}}}{\Delta Q_{\mathrm{total}}}∙100\%$$

The value of Δ*Q_Tx_* is the total thermal energy at a specific temperature. It can be obtained using Eq. (3) at the given temperature range. Therefore, the specific SoC at a specific temperature can be set in a better manner which makes the adjustment of the operating LTES system can be managed appropriately.

### Test tube

**Fig. 3:**
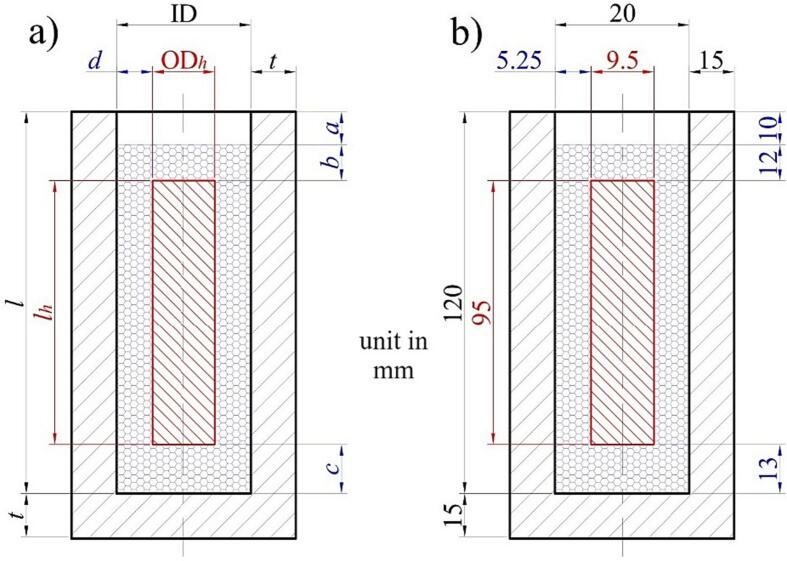
Geometry reference for test tube/cartridge heater (a) and example for detailed dimension in this work (b).

It is the only component that should be made manually (customized part). Determining the dimension of the test tube should be addressed carefully since it affects the heating rate and possible heat losses during the measurement. Fig. 3a shows the general configuration of the test tube and cartridge heater. The dimension of the test tube should be adjusted according to the heater dimension and the type of measured PCM. One critical aspect is the space between the heater and tube wall (*d*). If the distance is too wide, the heater cannot fully contact the sample, and the heat can be transferred to the tube wall if the distance is too small.

For reference, Fig. 3b displays the detailed dimension of the test tube in the present work. We use the PCM with an average density of 0.85 (relative to water density) for fatty acid and wax-based PCM. The wall thickness (*t*) is 15 mm to avoid heat transfer from the surrounding into the sample. The given dimension provides direct contact between the measured PCM and the heater. In addition, the dimension is flexible for readjustment based on the existing tube heater dimension.

### Instrumentation

**Fig. 4:**
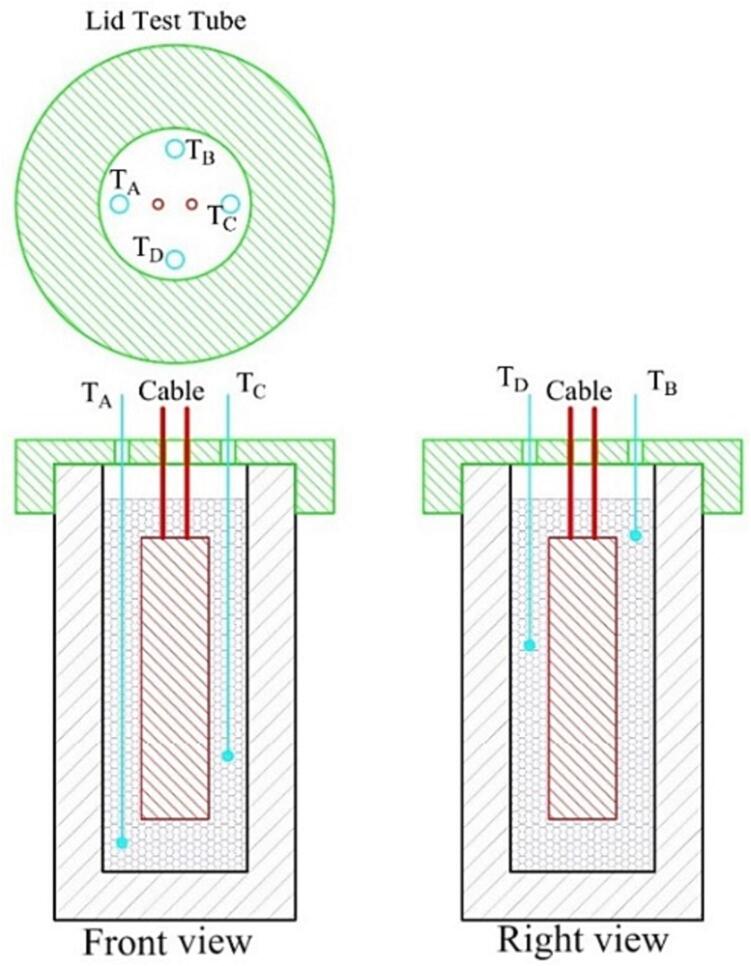
Temperature measurement location within the test tube.

As seen in Fig. 2, there are two direct measurements: temperature measurement of the sample within the test tube and electric energy supplied to the heater. The sample's temperature is measured in four different locations (Fig. 4). It is intended to monitor the temperature increment, reduce the measurement error and set the average temperature for data analysis. The melting process of PCM is a relative complex that involves expansion and contraction, which affect the molecular movement and shrinkage effect [23]. It demands multi-temperature measurements at different locations. We recommend measuring one location at the lower zone, two at the middle zone and one at the upper zone. A lid cover is specifically designed to ensure the precise position of the thermocouple probe within the test tube.

### Electrical circuit

**Fig. 5:**
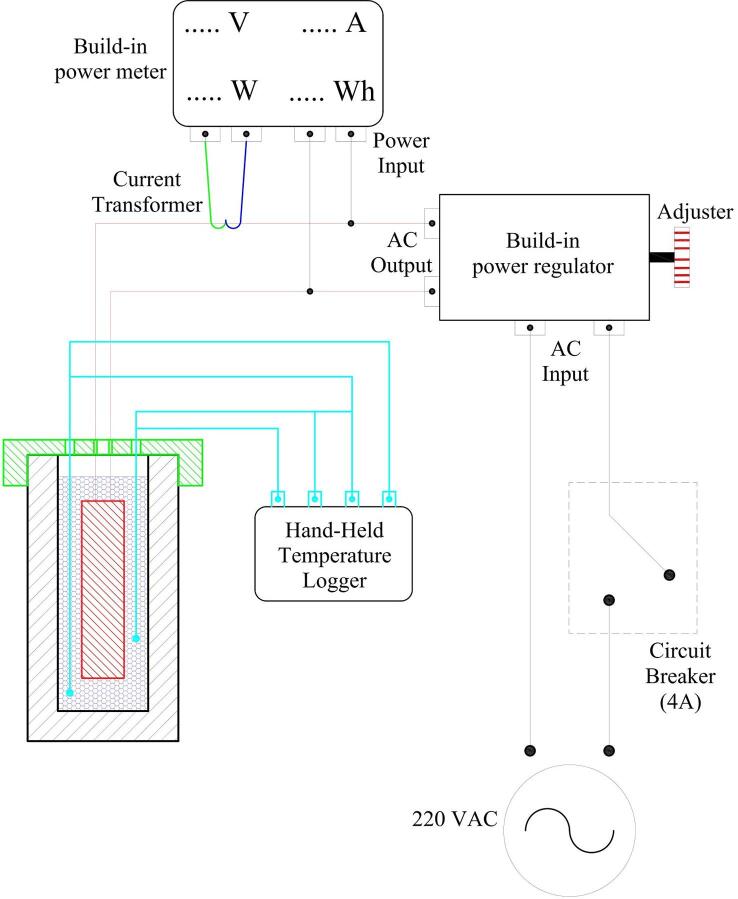
Wiring electrical diagram for the HETM apparatus.

Most of the components are built-in electrical components requiring no further adjustment. The electrical circuit is designed for alternating current (AC) of 220 V and 50 Hz. The detailed electrical circuit is presented in Fig. 5. We offer flexibility based on the availability of the components. The power meter can be replaced by an amperemeter and voltmeter logger version. The hand-held temperature logger can be switched into a computer-based temperature logger (Arduino version). The measurement device can be adjusted flexibly as long as the fundamental aspect can be achieved.

## Design files summary

The proposed HETM apparatus use built-in devices widely available in the marketplace. The only custom part is the test tube. A detailed drawing of the test tube is provided in the article. Therefore, there is no design file to build the HETM apparatus.

## Bill of materials summary

The currency rates during making the apparatus are Rp. 15,000/USD (Table 1). We offer flexibility for customization by following this condition:

a. Test tube: there is no strict rule for determining the material of the test tube. The key is low thermal conductivity material, good formability for machining, and higher melting temperature than the targeted measurement. Several materials, such as nylon, high-density polyethylene (HDPE) and polyoxymethylene (POM), can be used.
b. Temperature logger: user can use a hand-held model or an Arduino version.
c. Power meter: Optionally, it can be combined/replaced with a voltage and amperemeter logger.
d. Power regulator: analog model power control or programmable heater can be used to adjust the heating rate.

**Table 1:** Bills of material.

Designator	Component	Number	Cost per unit	Total cost	Source of materials	Material Type
Test tube	PTFE rod (outside diameter 50 mm, length 1.4 m)	1	$ 82.00	$ 82.00	Marketplace	Polytetrafluoroethylene (PTFE)
Temperature logger	Type–K, four channels, hand-held model, resolution: 0.1 °C, accuracy: ± 0.3 % + 1 °C	1	$ 300.00	$ 300.00	Marketplace	Other
Circuit breaker	Single phase, 220 V, 4 Ampere	1	$ 54.00	$ 54.00	Marketplace	Other
Power meter	Current transformer type, VAC, rating: 100A/22 kW, 45–65 Hz	1	$ 520.00	$ 520.00	Marketplace	Other
Thermocouple	Stick probe (outside diameter: 3 mm, length:300 mm), type K	4	$ 510.00	$ 540.00	Marketplace	Other
Power regulator	Pulse width modulation, VAC, rating power: 4 kW	1	$ 512.00	$ 512.00	Marketplace	Other
Cartridge heater	Model tube, outside diameter: 9.5 mm, length: 95 mm, power: 300 Watts	1	$ 520.00	$ 520.00	Marketplace	Stainless steel
Cable	14 AWG	20	$ 522.00	$ 544.00	Marketplace	Other
Connector	Banana jack (set), 6 mm	10	$ 521.00	$ 510.00	Marketplace	Other

## Build instructions

**Fig. 6:**
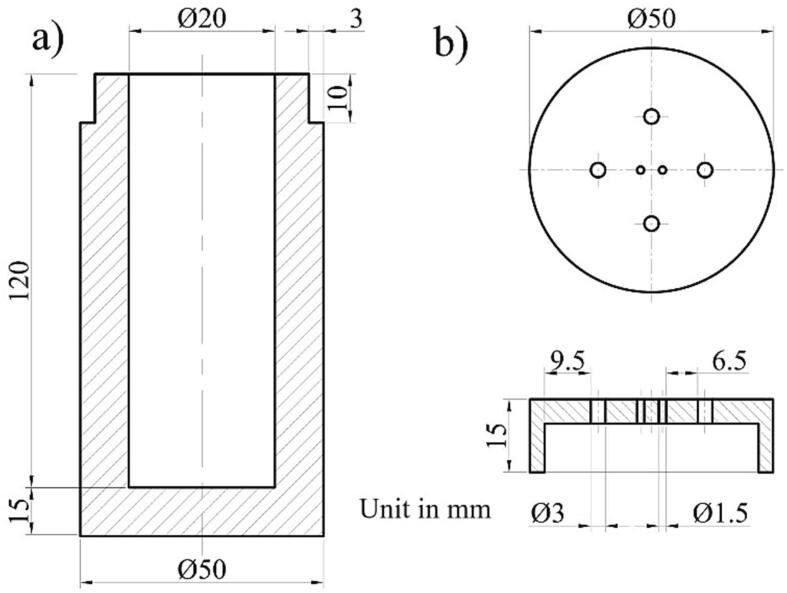
Detailed dimensions for test tube (a) and lid cover (b).
f. Start the machining process (we use a PTFE rod for the test tube). After that, measure the final test tube and lid cover. Then, clean the inside part of the test tube.
g. Measure the distance for locating the cartridge heater and four thermocouples within the test tube according to the initial configuration (Fig. 3). Set a suitable mark for the heater cable and thermocouples relative to the lid cover position.
h. Start the assembly process for all components and electrical circuits following the given diagram in Fig. 5. Also, there is no strict rule for locating the electrical component as long as the basic function for each component can be operated appropriately. Fig. 7 displays the final HETM apparatus.

The build instruction for the HETM apparatus is divided into two categories: manufacturing test tube and component assembly. The test tube is manufactured by following the procedure below:

a. Define the total mass of the evaluated sample. For example, we use 20 g of PCM for evaluation. After that, find the total volume of the PCM. We use the density ratio of 0.85 g/cm^3^ for the samples. Therefore, the volume of the PCM is 23.5 cm^3^ (∼24 cm^3^).
b. Find the dimension of the heater within the test tube. We recommend to find a high surface area with a minimum diameter to ensure a suitable heat distribution to the sample. We use a cartridge heater with a height of 95 mm and an outside diameter of 9.5 mm. The effective volume of the heater is ∼ 7 cm^3^.
c. Estimate the additional volume to accommodate the thermocouple probes and heater cable. We take an additional volume of 3 cm^3^.
d. Sum the total volume for the material, heater and additional volume (34 cm^3^). Then, add a volume tolerance of about + 10 % from the net volume to accommodate the expansion of the sample during phase transition. Finally, the total net volume for the test tube is 37.4 cm^3^ (∼37.5 cm^3^).
e. Fig. 6 presents the detailed design for the test tube and lid cover.

**Fig. 7:**
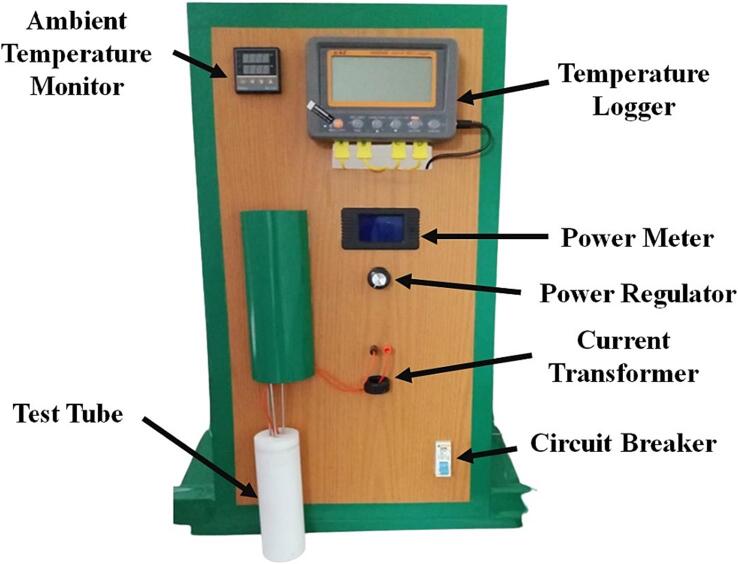
Final assembly of HETM apparatus.
i. Conduct an initial check for the electrical components, especially the power meter and regulator. Ensure the regulator able to set the targeted heating power.
j. Insert the heater into the test tube. Pull the heater cable out of the lid cover. Install the lid cover at the top of the test tube, then insert the four thermocouples into the tube through the lid cover. Ensure the height of the heater and thermocouples according to the defined position.

## Operation instructions

Ensure all component works properly before starting the measurement. The measurement is conducted as follows:

a. Locate the solid sample (20 g) in the beaker glass. Melt the sample at 80 °C using an electric oven or another heating device (for example, a hot plate).
b. Pour the molten sample into the test tube.
c. Insert the heater and thermocouples into the test tube according to the targeted height, then close the test tube with the lid cover.
d. Let the sample solidify at room temperature (30 °C).
e. During solidification, switch on the circuit breaker and adjust the power regulator to the targeted power rate (we use 10 Watts). After that, switch off the circuit breaker.
f. Once the sample is at room temperature, start the temperature recording process. Then, switch on the circuit breaker and let the heating process begin.
g. Record the measurement data (temperature and electric energy) until the sample reaches 80 °C. Then, stop the heating process (switch off the circuit breaker).
h. Plot the recorded data into the spreadsheet for initial analysis (Table 2).

**Table 2:** Recommended measurement spreadsheet (single measurement).

Time (second)	Energy (Joule)	Temperature (°C)				
		T_A_	T_B_	T_C_	T_D_	T_average_
i. To ensure the quality of the measurement, repeat the heating process five times. The measurement can be started from step (d).
j. Plot the measured energy and the average temperature increment of the sample from all measurements for final analysis (Table 3)

**Table 3:** Recommended spreadsheet format for final measurement.

Time (second)	Energy (Joule)						Temperature (°C)					
	*x_1_*	*x_2_*	*x_3_*	*x_4_*	*x_5_*	*x_final_*	*x_1_*	*x_2_*	*x_3_*	*x_4_*	*x_5_*	*x_final_*
k. After the final measurement, turn off all electrical and measurement components. Pull the heater and thermocouples slowly from the test tube while the sample is in the liquid phase (to avoid damage to the probes and heater surface).
l. Remove the solid sample from the test tube. Avoid using sharp/pointy tools during the removal to protect the inner wall of the test tube.
m. Pour hot oil at temperature 120 °C into the test tube for the final cleaning process. We recommend high-density oil (i.e., castor oil) to ensure the residual sample can be discharged entirely from the test tube.

## Validation and characterization

### Measurement error

The present work uses temperature in °C with an absolute error of thermocouple ± 0.2 °C. The power meter has a relative error 2.6 % since it uses current transformer. Theoretically, the electric heater has an efficiency closes to 100 % considering the fact that all electricity can be converted into usable heat. However, losses through the wiring system (cable and junction) are inevitable. Thus, we consider the usable heat from the electric heater is only 97 %. Another crucial aspect is the potential of heat losses to the test tube and environment. For this case, the average thermal conductivity of the PTFE and PCM are less than 1 W/m·K. It reduces the potential heat losses from the measurement. To minimize the issue, we highlighted to use small test tube as we used in this work. Also, the measurement is repeated to ensure the average value from each single measurement. Therefore, the risk of heat losses and measurement error can be neglected.

### Basic interpretation of thermal properties through HETM apparatus

**Fig. 8:**
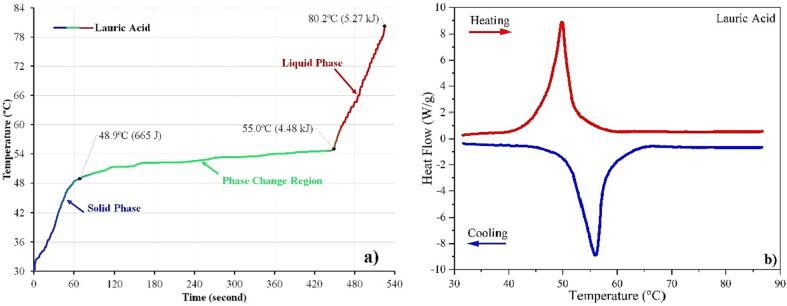
Profile temperature HETM measurement (a) and DSC curve (b) of lauric acid.

Interpreting measurement data is the critical aspect of using the HETM apparatus. We use lauric acid (dodecanoic acid) as an example for data interpretation from the measurement. Fig. 8a shows the heating curve of lauric acid from the HETM apparatus obtained from the average final measurement (Table 3). The profile indicates three distinguish heating curves: solid sensible, phase transition and liquid sensible. The change of temperature increment can be seen obviously from the profile temperature, which can be used to determine the transition area between the solid and liquid phases. Thus, the temperature for each phase can be obtained, which makes the thermal properties for solid and liquid phases can be analyzed using Eq. (1) and the energy balance from the power meter. The specific heat capacity for solid and liquid phases based on the temperature indicator is obtained at 1.765 J·g^−1^·°C^−1^ and 1.574 J·g^−1^·°C^−1^.

Fig. 8a demonstrates a slow temperature increment during the solid–liquid transition. It implies that the phase transition occurs non-isothermally and is accompanied by sensible heating simultaneously. It is the main feature for using the HETM apparatus to estimate precisely the sensible heating and latent heat of fusion during the solid–liquid transition. It implies that partial heat capacity should be considered from the absorbed heat energy during phase transition. For simplification, the partial heat capacity can be taken as the median value of solid and liquid heat capacity. Since the partial heat capacity is obtained, the latent heat of fusion can be estimated precisely using Eq. (3). Fig. 8b shows the DSC curve for lauric acid, which is found as a simple endothermic peak occurs at temperature 49.7 °C and melting enthalpy of 185.41 J·g^−1^.

Table 4 summarizes the melting temperature and enthalpy of lauric acid from the present work and previous studies. It shows various results for both properties. It implies the complexity of measuring the properties. Despite that, the melting temperature of lauric acid varies around + 3.6 °C insignificantly. The latent heat of fusion for the lauric acid ranges between 154.46 and 190 J·g^−1^. The melting temperature and enthalpy from the HETM apparatus is relatively close to the DSC measurement, which varies only 0.8 °C and 4.59 J·g^−1^. It confirms that the interpretation from the energy balance and temperature increment through the HETM apparatus can be taken as sufficient results to obtain the thermal properties of the lauric acid.

**Table 4:** Comparison of melting temperature and enthalpy of Lauric Acid.

Method	Melting temperature (°C)	Latent Heat of Fusion (J·g^−1^)	Ref
DSC	47.2	190.0	[24]
DSC	48.06	154.46	[25]
DSC	50.8	177.5	[26]
HETM	48.9	180.82	Present Work
DSC	49.7	185.41	

### Multiphase specific heat capacity and melting enthalpy of fatty acids PCM

**Fig. 9:**
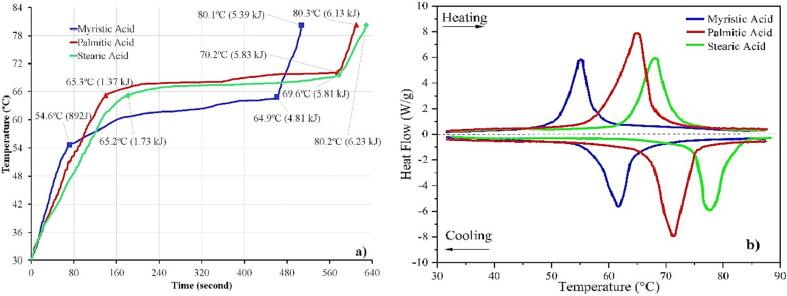
Profile temperature of fatty acids PCM from HETM measurement (a) and heating/cooling curve from DSC (b).

Three different fatty acids were characterized through the HETM apparatus. Fig. 9a shows a unique temperature profile for myristic, palmitic, and stearic acids. It demonstrates that each PCM has different thermal properties, making the heat capacity vary. The HETM apparatus allows more detailed observation of the thermal properties of each PCM. In agreement with the HETM profile, the DSC curve also signifies that each PCM has a unique endothermic curve at specific melting temperature even though it shows similar characteristics with a single solid–liquid peak (Fig. 9b).

**Fig. 10:**
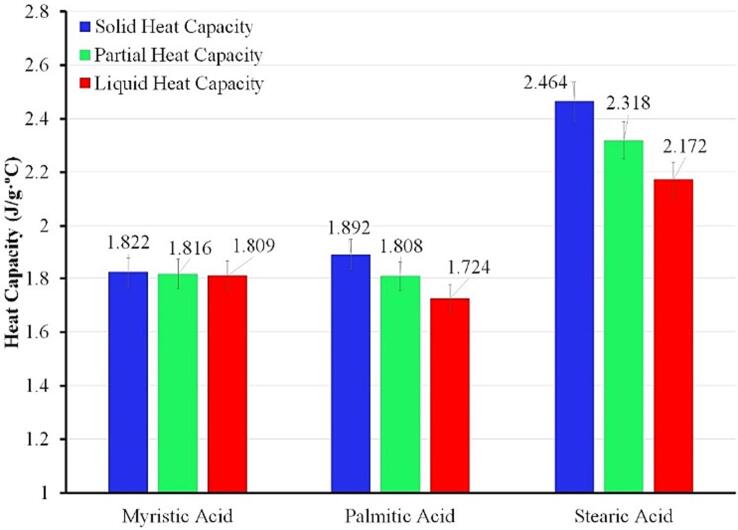
Detailed specific heat capacity for myristic, palmitic and stearic acid from HETM measurements.

The heat capacity for each phase, including partial heat capacity, is presented in Fig. 10. It shows that each fatty acid has a different heat capacity, making the temperature profile during measurement vary significantly. The heat capacity change for each phase is also affected by the nature of the material. For instance, the lowest solid heat capacity is obtained by myristic acid. It also has the lowest melting temperature (54.6 °C from the HETM apparatus and 55.5 °C from the DSC measurement).

Palmitic acid and stearic acid demonstrate a significant decrease in heat capacity at the liquid phase (11.3 % for palmitic acid and 11.8 % for stearic acid). It makes a rapid temperature increment in the liquid phase. Both samples also show a low-temperature gradient during phase transition, notably reducing partial heat capacity. In contrast, myristic acid shows unsubstantial heat capacity variation due to a high-temperature gradient during phase transition (10.3 °C). It signifies that the temperature-dependent thermophysical properties of each PCM should be taken into account for the actual application [27].

Table 5 compares previous studies' thermal properties of myristic acid, palmitic acid and stearic acid. Despite the discrepancy results, one can be seen that the variation of melting temperature and enthalpy show a unique characteristic. For example, the melting temperature for myristic acid ranges (+1.26 °C) insignificantly, while palmitic and stearic acid indicate a high variation for the melting temperature around + 9.84 °C and ± 15.49 °C, respectively. Contrary to that, the myristic acid has a high latent heat of fusion variation of around 28.69 J·g^−1^, while palmitic and stearic acid only 4.51 J·g^−1^ and 9.56 J·g^−1^, respectively. It is affected by each fatty acid's molecular weight and melting rate [37]. It proves the application of the HETM apparatus provides more detailed thermal properties which can be accompanied by DSC measurement for further material characterization and synthesis of new composite for LTES application.

**Table 5:** Comparison of melting temperature and enthalpy of myristic, palmitic and stearic acid.

PCM	Method	Melting temperature (°C)	Latent Heat of Fusion (J·g^−1^)	Ref
Myristic Acid (MA)	DSC	54.28	191.27	[28]
	DSC	54.5	169.7	[29]
	DSC	55.35	198.39	[30]
	HETM	54.6	178.3	Present Work
	DSC	55.5	193.64	
Palmitic Acid (PA)	DSC	59.66	209.35	[31]
	DSC	67.15	211.51	[32]
	DSC	69.5	213.86	[33]
	HETM	66.2	213.42	Present Work
	DSC	65.6	210.78	
Stearic Acid (SA)	DSC	52.9	191.6	[34]
	DSC	68.35	201.16	[35]
	DSC	68.39	200.5	[36]
	HETM	65.2	191.93	Present Work
	DSC	68.1	199.65	

### Multiphase specific heat capacity and melting enthalpy of wax-based PCM

**Fig. 11:**
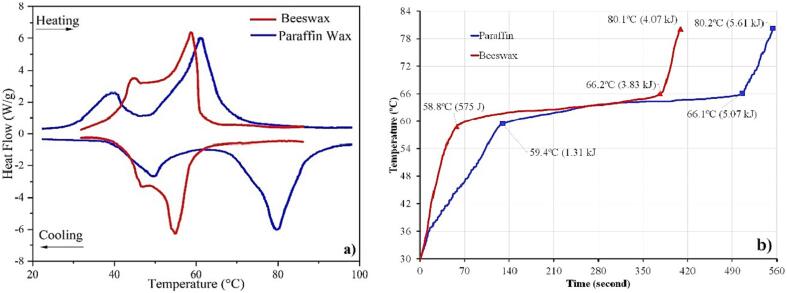
Heating/cooling curve from DSC for wax-based PCM (a) and profile temperature of paraffin and beeswax from HETM measurement (b).

Wax–based PCM (paraffin and beeswax) demonstrate a complex melting process compared to fatty acids. It can be seen from the appearance of the two consecutive endothermic curves (Fig. 11a). The first peak is defined as a solid–solid transition, while the second peak indicates the melting process.

It indicates that the solid transition is initiated before the melting process, which occurs at 58.9 °C (beeswax) and 61.3 °C (paraffin). The variation of the waxes' thermal properties causes different heating profiles from the HETM measurements (Fig. 11b).

**Fig. 12:**
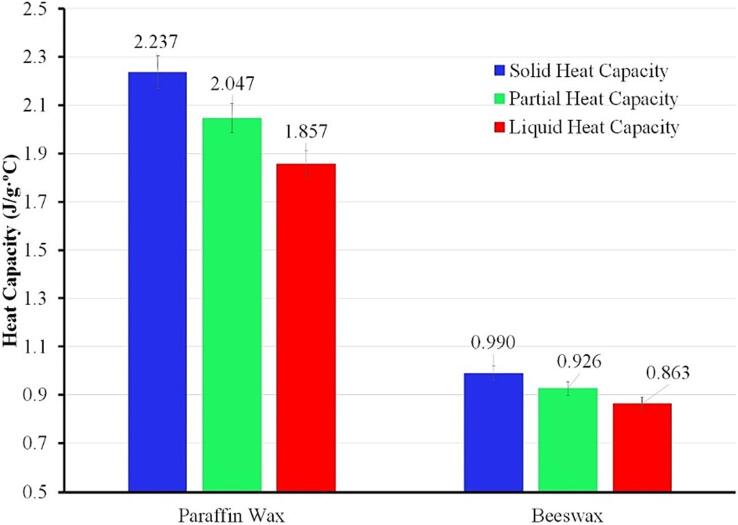
Detailed specific heat capacity for paraffin and beeswax.

As presented in Fig. 12, paraffin has a higher solid heat capacity compared to beeswax. It makes the paraffin requires more energy to reach the phase transition region, confirming the decrement after first endothermic peak (Fig. 11a). Despite that, both waxes indicate temperature gradient during phase transition, demonstrating the partial heat capacity during the melting process. The partial heat capacity for beeswax is relatively smaller than paraffin due to a low solid and liquid heat capacity. The decrement in the partial heat capacity is also unsubstantially caused by the high-temperature gradient during the solid–liquid transition (similar to myristic acid). The high decrement in the liquid heat capacity for paraffin causes a rapid increment during the liquid stage which can be observed notably from the heating profile (Fig. 11b).

Table 6 presents the melting temperature and enthalpy summary from previous studies for wax-based PCM. Both samples show an identic pattern where the melting temperature and enthalpy variation is generally higher than the fatty acids group. It can be affected by the purity level for each wax-based PCM. The variation for melting temperature is obtained at ± 7.61 °C and ± 5.28 °C while the latent heat of fusion varies between ± 42.94 J·g^−1^ and ± 60.8 J·g^−1^ for paraffin and beeswax, respectively.

**Table 6:** Comparison of melting temperature and enthalpy of wax-based PCM.

PCM	Method	Melting temperature (°C)	Latent Heat of Fusion (J·g^−1^)	Ref
Paraffin Wax	DSC	53.29	162.3	[38]
	DSC	59.07	137.4	[39]
	DSC	61.3	179.2	[40]
	HETM	59.4	174.28	Present Work
	DSC	58.9	180.34	
Beeswax	DSC	57.0	153.2	[41]
	DSC	59.8	214	[42]
	DSC	62.28	141.9	[43]
	HETM	58.8	156.14	Present Work
	DSC	58.9	171.66	

The high variation for the wax-based PCM signifies the presence of solid–solid transition from the DSC curve (Fig. 12a). It makes the measurement of heat capacity for paraffin and beeswax becomes more complex than fatty acids. The HETM apparatus offers a reliable measurement that can be useful for assessing the detailed thermal properties of metastable PCM material, such as paraffin and beeswax.

### SoC estimation from HETM measurements

**Fig. 13:**
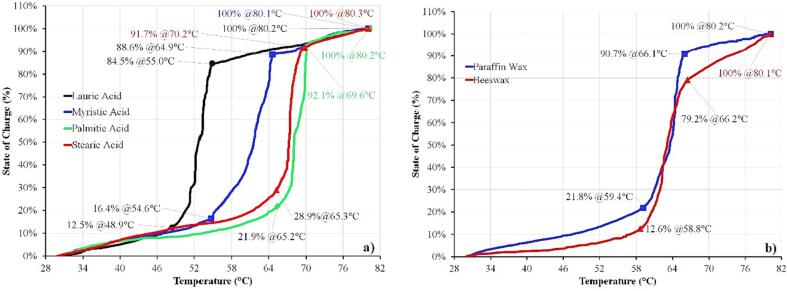
Specific SoC percentage at a specific temperature for fatty acids (a) and wax-based PCM (b).

The estimation of total heat capacity at the three-stage operation of solid–liquid PCM allows to set a precise SoC estimation based on the given temperature using Eq. (4). The specific SoC percentage at the given temperature range from the HETM measurement is plotted in Fig. 13. All samples indicate one specific character that the highest SoC percentage is obtained during phase transition. The solid and liquid sensible contributes around 28 % – 37.2 % (fatty acids) and 31.1 % – 33.4 % (wax-based PCM) from the total stored heat. It makes one significant advantage for the operation of the LTES system where the highest energy can be absorbed and released during the phase change that contributes more than 60 %.

The HETM apparatus allows for estimating the specific SoC rate during the solid–liquid transition. For instance, the temperature gradient for lauric acid is 6.1 °C with 72 % SoC percentage during phase transition. Thus, the specific SoC rate for lauric acid is obtained at 11.8 %/°C. The highest specific SoC rate is obtained by palmitic acid with 15.9 %/°C. It is affected by the low-temperature gradient (4.4 °C) and high SoC percentage during the solid–liquid transition (70.2 %). The value is essential for setting the charging/discharging protocol of the LTES system and ensuring the effective volumetric energy ratio [44]. Also, it is essential for material development since the lowest temperature gradient is desirable for the LTES system. The specific SoC rate is beneficial for both material development and LTES operation, making the HETM apparatus favorable for estimating the total heat capacity of low-temperature multiphase latent thermal energy storage material.

### Composite and immiscible PCM

Further evaluation was performed using composite PCM and HDPE. The HDPE is reliable to promote a better phase transition process for the organic PCM. The HETM apparatus was used for initial evaluation and combined with DSC measurement and active test. The finding indicated that the supercooling of PCM can be reduced up to 4.2 °C. Also, the composites show a higher specific SoC during phase transition. Both PCMs categories were evaluated through active test and showed the composite has a higher storage efficiency around 90.2 % than pure PCM with only 77.1 %. The detailed results can be seen in this reference [45].

Another evaluation was taken using inorganic salt (ternary mixture). The salt is mixed with HDPE to promote a direct contact immiscible PCM. The basic concept of HETM was employed for characterizing the performance of the system. It showed the multistep profile of the mixture can be characterized specifically. Also, the phase behavior during phase transition can be evaluated distinctively where the mixture has a temperature gradient 13.9 °C. The proposed approach was a preliminary characterization in order to develop a better direct contact PCM for medium temperature application. The finding from the work can be seen in this reference [46].

## Declaration of Competing Interest

The authors declare that they have no known competing financial interests or personal relationships that could have appeared to influence the work reported in this paper.

## References

[1] IRENA (2020). Innovation Outlook. Thermal Energy Storage.

[2] Favakeh A., Khademi A., Shafii M.B. (2019). 7th International Conference On Energy Research and Development, ICERD 2019.

[3] Hosseininaveh H., Rahgozar Abadi I., Mohammadi O., Khademi A., Behshad Shafii M. (2022). The impact of employing carbon nanotube and Fe3O4 nanoparticles along with intermediate boiling fluid to improve the discharge rate of phase change material. Appl. Therm. Eng..

[4] Khademi A., Mehrjardi S.A.A., Said Z., Chamkha A.J. (2023). Heat transfer improvement in a thermal energy storage system using auxiliary fluid instead of nano-PCM in an inclined enclosure: A comparative study. J. Appl. Comput. Mech..

[5] Khademi A., Mehrjardi S.A.A., Said Z., Saidur R., Ushak S., Chamkha A.J. (2023). A comparative study of melting behavior of phase change material with direct fluid contact and container inclination. Energy Nexus..

[6] Couvreur K., Beyne W., Tassenoy R., Lecompte S., De Paepe M. (2023). Characterization of a latent thermal energy storage heat exchanger using a charging time energy fraction method with a heat loss model. Appl. Therm. Eng..

[7] Suyitno B.M., Ismail I., Rahman R.A. (2023). Improving the performance of a small-scale cascade latent heat storage system by using gradual melting temperature storage tank. Case Studies Thermal Eng..

[8] Ismail I., Syahbana M.S.L., Rahman R.A. (2022). Thermal performance assessment for an active latent heat storage tank by using various finned-coil heat exchangers. Int. J. Heat Technol..

[9] A. Khademi, M. Darbandi, G.E. Schneider, Numerical study to optimize the melting process of phase change material coupled with extra fluid, AIAA Scitech 2020 Forum. 1 PartF (2020) 1–6. 10.2514/6.2020-1932.

[10] Fatahi H., Claverie J., Poncet S. (2022). Thermal characterization of phase change materials by differential scanning calorimetry: A review. Appl. Sci. (Switzerland)..

[11] Rolka P., Kwidzinski R., Przybylinski T., Tomaszewski A. (2021). Thermal characterization of medium-temperature phase change materials (Pcms) for thermal energy storage using the t-history method. Materials.

[12] Brütting M., Vidi S., Hemberger F., Ebert H.P. (2019). Dynamic T-History method - A dynamic thermal resistance for the evaluation of the enthalpy-temperature curve of phase change materials. Thermochim. Acta.

[13] Tan P., Brütting M., Vidi S., Ebert H.P., Johansson P., Sasic Kalagasidis A. (2018). Characterizing phase change materials using the T-History method: On the factors influencing the accuracy and precision of the enthalpy-temperature curve. Thermochim. Acta.

[14] Bastida H., De la Cruz-Loredo I., Ugalde-Loo C.E. (2023). Effective estimation of the state-of-charge of latent heat thermal energy storage for heating and cooling systems using non-linear state observers. Appl. Energy.

[15] Barz T., Seliger D., Marx K., Sommer A., Walter S.F., Bock H.G., Körkel S. (2018). State and state of charge estimation for a latent heat storage. Control Eng. Pract..

[16] Reboli T., Ferrando M., Traverso A., Chiu J.N.W. (2022). Thermal energy storage based on cold phase change materials: Charge phase assessment. Appl. Therm. Eng..

[17] Hashem Zadeh S.M., Ghodrat M., Ayoubi Ayoubloo K., Sedaghatizadeh N., Taylor R.A. (2022). Partial charging/discharging of bio-based latent heat energy storage enhanced with metal foam sheets. Int. Commun. Heat Mass Transfer.

[18] Dauvergne J.L., Serrano Á., Palomo Del Barrio E. (2021). Fast estimation of the enthalpy–temperature function of Phase Change Materials. Exp. Therm Fluid Sci..

[19] Favakeh A., Khademi A., Shafii M.B. (2023). Experimental investigation of the melting process of immiscible binary phase change materials. Heat Transfer Eng..

[20] Pandey K., Ali S.F., Gupta S.K., Saikia P., Rakshit D., Saha S. (2021). Facile technique to encapsulate phase change material in an amphiphilic polymeric matrix for thermal energy storage. Appl. Energy.

[21] Barz T. (2021). Paraffins as phase change material in a compact plate-fin heat exchanger - Part II: Validation of the “curve scale” hysteresis model for incomplete phase transitions. J. Storage Mater..

[22] Rahman R.A., Lahuri A.H., Ismail I. (2023). Thermal stress influence on the long-term performance of fast-charging paraffin-based thermal storage. Thermal Sci. Eng. Prog..

[23] Janghel D., Saha S.K., Karagadde S. (2020). Effect of shrinkage void on thermal performance of pure and binary phase change materials based thermal energy storage system: A semi-analytical approach. Appl. Therm. Eng..

[24] Cao X., Zhang R., Zhang N., Chen L., Chen D., Li X. (2023). Performance improvement of lauric acid-1-hexadecanol eutectic phase change material with bio-sourced seashell powder addition for thermal energy storage in buildings. Constr. Build. Mater..

[25] Alkhazaleh A.H., Almanaseer W., Alkhazali A. (2023). Experimental investigation on thermal properties and fire performance of lauric acid/diphenyl phosphate/expanded perlite as a flame retardant phase change material for latent heat storage applications. Sustainable Energy Technol. Assess..

[26] Chauhan V.K., Shukla S.K. (2023). Performance analysis of Prism shaped solar still using Black phosphorus quantum dot material and Lauric acid in composite climate : An experimental investigation Conventional Solar still Prism type Solar Still Computational Fluid dynamics. Sol. Energy.

[27] Li Z., Gariboldi E. (2021). Review on the temperature-dependent thermophysical properties of liquid paraffins and composite phase change materials with metallic porous structures. Mater. Today Energy.

[28] He Q., Fei H., Zhou J., Du W., Pan Y., Liang X. (2022). Preparation and characteristics of lauric acid-myristic acid-based ternary phase change materials for thermal storage. Mater. Today Commun..

[29] Mandal S., Ishak S., Lee D.E., Park T. (2022). Shape-stabilized orange peel/myristic acid phase change materials for efficient thermal energy storage application. Energy Rep..

[30] Li C., Li Q., Ge R., Lu X. (2023). A novel one-step ultraviolet curing fabrication of myristic acid-resin shape-stabilized composite phase change material for low temperature thermal energy storage. Chem. Eng. J..

[31] Zhang R., Chen D., Chen L., Cao X., Li X., Qu Y. (2022). Preparation and thermal properties analysis of fatty acids/1-hexadecanol binary eutectic phase change materials reinforced with TiO2 particles. J. Storage Mater..

[32] Konuklu Y., Akar H.B. (2023). Promising palmitic acid/poly(allyl methacrylate) microcapsules for thermal management applications. Energy.

[33] Lee W., Kim J. (2022). Poly(dimethylsiloxane) grafting on palmitic acid and surface of expanded graphite for advanced phase change material composites. J. Storage Mater..

[34] Zhao X., Li C., Bai K., Xie B., Chen J., Liu Q. (2022). Multiple structure graphite stabilized stearic acid as composite phase change materials for thermal energy storage, International Journal of. Min. Sci. Technol..

[35] Ao C., Yan S., Zhao S., Hu W., Zhao L., Wu Y. (2022). Stearic acid/expanded graphite composite phase change material with high thermal conductivity for thermal energy storage. Energy Rep..

[36] Fang G., Zhao M., Sun P. (2022). Experimental study of the thermal properties of a fatty acid-modified graphite composite phase change material dispersion system. J. Storage Mater..

[37] Al-Ahmed A., Mazumder M.A.J., Salhi B., Sari A., Afzaal M., Al-Sulaiman F.A. (2021). Effects of carbon-based fillers on thermal properties of fatty acids and their eutectics as phase change materials used for thermal energy storage: A Review. J. Storage Mater..

[38] Zhang Y., Feng D., Liu Q., Wang X., Li K., Su J., Zhang X., Feng Y. (2022). Paraffin wax with an addition of nano Ti2O3: Improve thermal and photothermal performances with little decreased latent heat. Sol. Energy.

[39] Hou Y., Chen H., Liu X. (2022). Experimental study on the effect of partial filling of copper foam on heat storage of paraffin-based PCM. Renew. Energy.

[40] Suyitno B.M., Rahmalina D., Rahman R.A. (2023). Increasing the charge / discharge rate for phase-change materials by forming hybrid composite paraffin / ash for an effective thermal energy storage system. AIMS Material Science..

[41] Putra N., Rawi S., Amin M., Kusrini E., Kosasih E.A., T.m. (2019). Indra Mahlia, Preparation of beeswax/multi-walled carbon nanotubes as novel shape-stable nanocomposite phase-change material for thermal energy storage. J. Storage Mater..

[42] Dinker A., Agarwal M., Agarwal G.D. (2017). Experimental assessment on thermal storage performance of beeswax in a helical tube embedded storage unit. Appl. Therm. Eng..

[43] Amin M., Putra N., Kosasih E.A., Prawiro E., Luanto R.A., Mahlia T.M.I. (2017). Thermal properties of beeswax/graphene phase change material as energy storage for building applications. Appl. Therm. Eng..

[44] Ali S., Mehrjardi A., Khademi A., Said Z. (2023). Enhancing latent heat storage systems: the impact of PCM volumetric ratios on energy storage rates with auxiliary fluid assistance. Energy Nexus.

[45] Suyitno B.M., Pane E.A., Rahmalina D., Rahman R.A. (2023). Improving the operation and thermal response of multiphase coexistence latent storage system using stabilized organic phase change material. Results Eng..

[46] Muliawan B., Anggrainy R., Plamonia N., Rahman R.A. (2023). Preliminary characterization and thermal evaluation of a direct contact cascaded immiscible inorganic salt / high-density polyethylene as moderate temperature heat storage material. Results Mater..

